# Comparative Characterization of *Pseudoroegneria libanotica* and *Pseudoroegneria tauri* Based on Their Repeatome Peculiarities

**DOI:** 10.3390/plants12244169

**Published:** 2023-12-15

**Authors:** Pavel Yu. Kroupin, Anna I. Yurkina, Daniil S. Ulyanov, Gennady I. Karlov, Mikhail G. Divashuk

**Affiliations:** 1 All-Russia Research Institute of Agricultural Biotechnology, Timiryazevskaya St., 42, 127550 Moscow, Russiauldas1508@gmail.com (D.S.U.);; 2 Federal Research Center “Nemchinovka”, Bolshoi Blvd., 30 Bld. 1, Skolkovo Innovation Center, 121205 Moscow, Russia; 3 National Research Center “Kurchatov Institute”, Kurchatov Sq., 1, 123182 Moscow, Russia

**Keywords:** fluorescence in situ hybridization, *Pseudoroegneria*, St genome, satellite repeats, shallow whole-genome sequencing

## Abstract

*Pseudoroegneria* species play an important role among *Triticeae* grasses, as they are the putative donors of the St genome in many polyploid species. Satellite repeats are widely used as a reliable tool for tracking evolutionary changes because they are distributed throughout the genomes of plants. The aim of our work is to perform a comparative characterization of the repeatomes of the closely related species *Ps. libanotica* and *Ps. tauri*, and *Ps. spicata* was also included in the analysis. The overall repeatome structures of *Ps. libanotica*, *Ps. tauri*, and *Ps. spicata* were similar, with some individual peculiarities observed in the abundance of the *SIRE* (Ty1/*Copia*) retrotransposons, *Mutator* and *Harbinger* transposons, and satellites. Nine new satellite repeats that have been identified from the whole-genome sequences of *Ps. spicata* and *Ps. tauri*, as well as the CL244 repeat that was previously found in *Aegilops crassa*, were localized to the chromosomes of *Ps. libanotica* and *Ps. tauri*. Four satellite repeats (CL69, CL101, CL119, CL244) demonstrated terminal and/or distal localization, while six repeats (CL82, CL89, CL168, CL185, CL192, CL207) were pericentromeric. Based on the obtained results, it can be assumed that *Ps. libanotica* and *Ps. tauri* are closely related species, although they have individual peculiarities in their repeatome structures and patterns of satellite repeat localization on chromosomes. The evolutionary fate of the identified satellite repeats and their related sequences, as well as their distribution on the chromosomes of *Triticeae* species, are discussed. The newly developed St genome chromosome markers developed in the present research can be useful in population studies of *Ps. libanotica* and *Ps. tauri*; auto- and allopolyploids that contain the St genome, such as *Thinopyrum*, *Elymus*, *Kengyilia*, and *Roegneria*; and wide hybrids between wheat and related wild species.

## 1. Introduction

The genus *Pseudoroegneria* (Nevski) A. Löve consists mainly of cool-season grasses that are distributed in the Middle East, central Asia, Transcaucasia, northern China, and western North America [1]. Representatives of this genus are distinguished by their significant ecological plasticity and their ability to survive in arid steppe conditions [2]. They also possess excellent forage quality [1,3,4,5]. *Pseudoroegneria* evolved 14.4–14.7 million years ago, making it more ancient than *Triticum*/*Aegilops* (8.0–8.3 Myr) [6]. *Pseudoroegneria* is represented by approximately 15 different species, including six diploids and nine autotetraploids. These species contain more than one variant of the St genome which suggests their polyphyletic origin [7,8].

*Pseudoroegneria* species are carriers of the St genome, which holds a unique position among *Triticeae* species. Cytogenetic studies using genomic in situ hybridization and the comparative characterization of the EST-SSR and rDNA ITS sequences and single-copy nuclear genes have shown that the St subgenome in allopolyploid species *Elytrigia*, *Elymus*, *Thinopyrum*, *Kengyilia*, and *Roegneria* most likely originated from different *Pseudoroegneria* donors [9,10,11,12,13,14,15,16,17,18,19,20,21]. A characterization of genes and the intergenic regions of the chloroplast and mitochondrial genomes demonstrated that *Pseudoroegneria* is the most likely (or one of the most likely) maternal donor in the allopolyploid species of *Elymus*, *Thinopyrum*, *Kengyilia*, and *Roegneria* [8,9,11,16,22,23,24,25,26,27,28,29,30,31,32,33,34]. Interestingly, the St subgenome of the same allopolyploid species may differ among different populations. This variation could be attributed to their polyphyletic origin and reticulate evolution [8,30]. Parental diversity and heterogeneity may be the reasons why the *Pseudoroegneria* species became a central maternal donor in *Triticeae*. Their genetic diversity provides the basis for adaptability and enhances the fitness of their descendants [8].

At the same time, inconsistencies often occur between phylogenetic trees constructed using different genes, primarily due to incomplete lineage sorting, chloroplast captures, nuclear gene exchange through hybridization, and subsequent introgressions [8,35]. These conflicts can be partially resolved by using whole-genome sequencing data as input for comparative characterization and phylogenetic analyses. With the emergence of whole-genome sequencing technologies, it has become feasible to conduct a comprehensive analysis of *Triticeae* genomes and determine the phylogenetics of *Pseudoroegneria* through a comparative analysis of the nuclear genome [5,36], chloroplast genomes [8,37], and transcriptomes [6].

Repeated elements are a reliable tool for tracking evolutionary change because they are widely distributed throughout the genome. These include both mobile elements and satellite repeats, both dispersed and tandem. They are widely used for karyotyping chromosomes, studying chromosomal rearrangements, and analyzing the genomic composition of allo- and autopolyploids and wide hybrids using fluorescence in situ hybridization (FISH) [38,39,40,41,42]. Comparative characteristics between *Triticeae* species can be studied by comparing the copy numbers of repeating elements [43], by comparing the distribution patterns across chromosomes and genomes [44,45,46,47], or by using a combination of both approaches [48,49].

Owing to the development of whole-genome sequencing technologies and bioinformatics analysis algorithms, it has become possible to quickly and efficiently create new chromosomal markers based on satellite repeats [50,51].

*Ps. libanotica* and *Ps. tauri* are closely related species that grow in Central Asia, specifically in Turkey, Iraq, Iran, and Syria. They are distinct from other *Pseudoroegneria* species as they have no awns with unequal glumes [52]. The similarity of their genomes was demonstrated by analyzing chromosome pairing in interspecific hybrids [53], spectra of glutenins and gliadins [54], chloroplast and single-copy nuclear genes [55,56,57], complete chloroplast genomes [37], and Pong-like transposase sequences [58].

The comparative characteristics of closely related species are of interest for studying both the divergence of the St genome itself, which is central to a significant number of species, and for understanding the evolutionary processes within the *Triticeae* tribe. Here, a comparative analysis of two closely related species, *Ps. libanotica* and *Ps. tauri*, was performed by comparing their repeatomes and characterizing the chromosomal localization of newly discovered St-genome satellite repeats.

## 2. Results

### 2.1. Repeatome Characterization

The repeatome structures of *Ps. libanotica*, *Ps. tauri*, and *Ps. spicata* are shown in Table 1 and Figures S1–S3. The repeatome of *Ps. libanotica*, *Ps. tauri*, and *Ps. spicata* was mostly represented by mobile elements (37.62%, 37.24%, and 43.05%, respectively; hereafter, percentages refer to these species in this order), most of which are retrotransposons (35.31%, 35.01%, and 40.03%), followed by DNA transposons (2.31%, 2.23%, and 3.02%). Ty3/*Gypsy* elements (20.98%, 21.99%, and 24.58%) were more abundant than Ty1/*Copia* elements (7.92%, 8.34%, and 9.30%) in the studied species. The most common Ty3/*Gypsy* elements were *Athila* (10.35%, 12.31%, and 11.69%) and *Tekay* (5.89%, 5.37%, and 6.42%). The Ty1/*Copia* superfamily elements were mainly composed of the *Angela* (4.85%, 5.33% and 4.64%) and *SIRE* (2.71%, 2.77%, and 4.41%) lineages. In *Ps. spicata*, the proportion of Ty1/*Copia* is higher compared to *Ps. libanotica* and *Ps. tauri* due to the higher *SIRE* abundance. Among transposons, the most frequent lineage appeared to be *CACTA* (2.00%, 1.95%, and 2.75%). Also, *Ps. spicata* is characterized by a higher abundance of *Mutator* transposons (0.25%) compared to *Ps. libanotica* (0.18%) and *Ps. tauri* (0.07%). *Harbinger* transposons in *Ps. spicata*, on the contrary, are present in a smaller proportion, 0.02%, compared to 0.12% in *Ps. libanotica* and 0.21% in *Ps. tauri*. The satellites were more abundant in *Ps. libanotica* (5.35%) and *Ps. spicata* (5.42%) compared to *Ps. tauri* (2.36%).

### 2.2. Satellite Repeats Characterization and Their Chromosomal Localization in Ps. libanotica and Ps. tauri

The satellite repeats CL89, CL185, and CL192 were found in the *Ps. tauri* genome, while CL69, CL82, CL101, CL119, CL168, and CL207 were identified in the *Ps. spicata* genome. The CL244 repeat, which we had previously discovered in the *Aegilops crassa* genome [51], was also utilized in the experiments of in situ hybridization. For convenience, here we first describe repeats with terminal or distal localization (CL69, CL101, CL119, and CL244) and then those with mainly pericentromeric localization (CL82, CL89, CL168, CL185, CL192, and CL207). The identified repeats were submitted to the NCBI GenBank system, and the IDs OR800789-OR800793, OR800795, OR800800-OR800802 were obtained.

**Figure 1:**
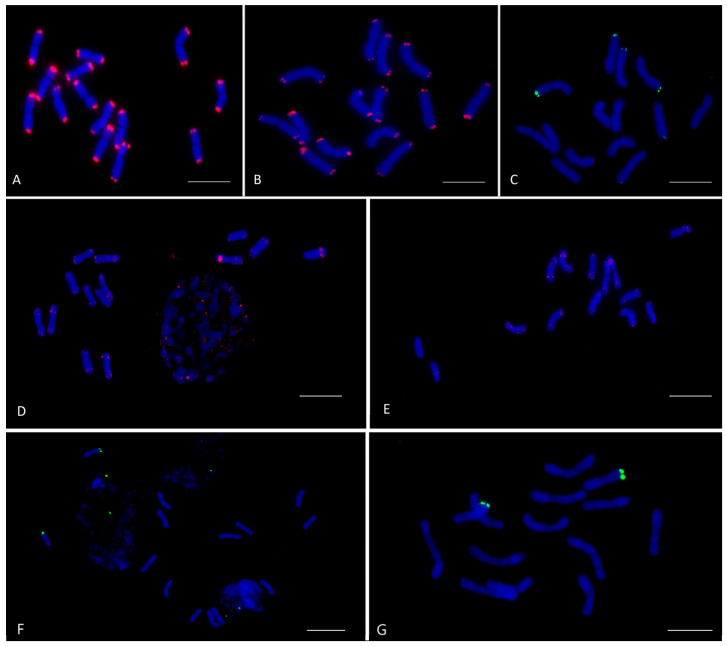
Chromosomal localization of satellite repeats CL69 (**A**,**B**), CL101 (**C**), CL119 (**D**,**E**), and CL244 (**F**,**G**) on metaphase cells of *Ps. libanotica* (**A**,**D**,**F**) and *Ps. tauri* (**B**,**C**,**E**,**G**) using fluorescence in situ hybridization. CL101 and CL244 were labeled digoxigenin-11-dUTP (green), CL69 and CL119—biotin-16-dUTP (red). Chromosomes counterstained with DAPI (blue). The bar indicates 10 µm.

CL69. CL69 has a length of 178 bp and a 0.377% genome proportion. It shared a 98.2% identity with oligo-7E-744 from *Thinopyrum elongatum*, a 92.4% identity with oligo-6VS-57 from *Dasypyrum villosum*, an 82.4% identity with CL239 from *Ae. crassa*, and a 71.9% identity with CL211 from *Th. bessarabicum* (Table 2 and Table S1). In both studied *Pseudoroegneria* species, CL69 is localized terminally, but the signals appear stronger on the chromosomes of *Ps. libanotica*. In all fourteen chromosomes of *Ps. libanotica,* the signals are terminal and localized to both arms. The CL69 hybridization in *Ps. tauri* differs from that in *Ps. libanotica* not only by signal intensity but also by the absence of a hybridization site on the long arm of one chromosome (Figure 1).

CL101. CL101 has a length of 177 bp and a 0.253% genome proportion. It shared a 79.5% identity with oligo-7E-744 from *Th. elongatum*, a 68–76% identity with the Spelt-1 and Spelt1-similar telomeric repeats pSp1B16 and Tri-MS-6, and a 71.4% identity with CL239 from *Ae. crassa* (Table 2 and Table S1). Six chromosomes of *Ps. tauri* carry terminal signals of CL101: four chromosomes showed signals on the short arm, while two chromosomes showed signals on the long arm. The strongest signal is observed on one chromosome, while the rest are very faint. In the chromosomes of *Ps. libanotica* CL101 signals are absent (Figure 1).

CL119. CL119 has a length of 668 bp and a 0.209% genome proportion. It shared a 94,7% identity with CL232 from *Ae. crassa*, a 90% identity with Olgo-1AL from *T. aestivum*, and an 84.9–89.7% identity with variants of BSCL156 from *Th. bessarabicum*.

Additionally, identity in the range 74–86.9% was shown (in descending order) with 18–158 from *Th. ponticum*, CL149 from *Th. bessarabicum*, pAcPR5 from *Agropyron cristatum*, CL131 from *Ae. crassa*, the pTa-465 clone from *Triticum aestivum*, AesTR-183 from *Ae. speltoides*, and Sc26c38 from *Secale cereale* (Table 2 and Table S1). In the studied *Pseudoroegneria* species, CL119 predominantly produces minor signals in the terminal and distal regions of most chromosomes. In two chromosomes of *Ps. libanotica*, intense CL119 signals are observed in the distal part of the long arm. In *Ps. tauri*, distinct distal CL119 signals are observed on the short arm of two chromosomes. In addition, minor signals are observed in the distal, interstitial, and proximal regions on other chromosomes in both species (Figure 1).

CL244. In both *Ps. libanotica* and *Ps. tauri*, two chromosomes carry terminal hybridization sites of CL244 on the long arm (Figure 1).

**Figure 2:**
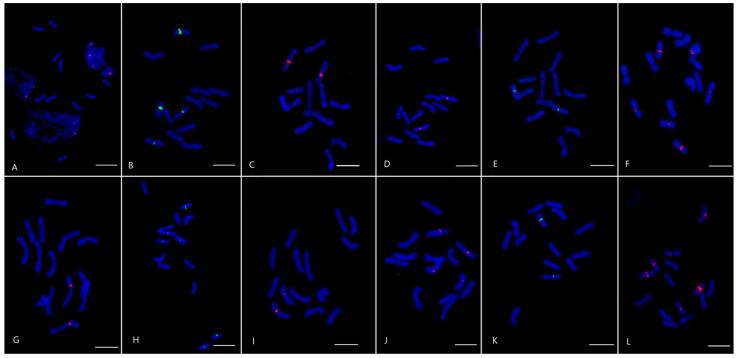
Chromosomal localization of satellite repeats CL82 (**A**,**G**), CL89 (**B**,**H**), CL168 (**C**,**I**), CL185 (**D**,**J**), CL192 (**E**,**K**), and CL207 (**F**,**L**) on metaphase cells of *Ps. libanotica* (**A**–**F**) and *Ps. tauri* (**G**–**L**) using fluorescence in situ hybridization. CL89 and CL192 were labeled digoxigenin-11-dUTP (green), CL82, CL168, CL185 and CL207—biotin-16-dUTP (red). Chromosomes counterstained with DAPI (blue). The bar indicates 10 µm.

CL82. CL82 has a length of 503 bp and a 0.335% genome proportion. It shared an 88% identity with the clone pTa-451 from T. aestivum and an 85% identity with CL18 from *Ae. crassa* and P631 from *Ae. tauschii*. Additionally, a lower identity (75–85%) was found for CL3 from *Ae. crassa*, the FAT element, oligo-5D151 from *T. aestivum*, StLIB98 from *Ps. libanotica*, oligo-7E-430 from *Th. elongatum*, and P523 from *Ae. tauschii* (Table 3 and Table S2). The CL82 signals are located pericentromerically on the two chromosomes, both in *Ps. libanotica* and *Ps. tauri* (Figure 2).

CL89. CL89 has a length of 658 bp and a 0.241% genome proportion. It shared a 100% identity with P631 from *Ae. tauschii*. In addition, identity in the range 75–90% was found for the pAs1 oligos and clones, P720 from *Ae. tauschii*, and CL3, CL193, and CL18 from *Ae. crassa* (Table 3 and Table S2). CL89 has a similar signal distribution pattern in *Ps. libanotica* and *Ps. tauri*. Pericentromeric signals of CL89 are localized to six chromosomes of *Ps. tauri* and four chromosomes of *Ps. libanotica* (Figure 2).

CL168. CL168 has a length of 476 bp and a 0.070% genome proportion. It shared a 91.7% identity with CL18 from *Ae. crassa* and the FAT element. A lesser degree (75–90%) was observed for P631 from *Ae. tauschii*, CL193 from *Ae. crassa*, CL80 from *A. cristatum*, and CL148 from *Th. bessarabicum* (Table 3 and Table S2). CL168 is localized pericentromerically to two *Ps. tauri* chromosomes, and while the signal on one chromosome is bright, on the second it is minor. In *Ps. libanotica*, large pericentromeric signals are observed on two chromosomes, and minor pericentromeric and interstitial signals on the remaining chromosomes are visible (Figure 2).

CL185. CL185 has a length of 659 bp and a 0.033% genome proportion. It shared a 95.4% identity with P631 from *Ae. tauschii* and a 91.7% identity with CL18 from *Ae. crassa*. Additionally, identity in the range 74–83% was shown for the FAT element, CL193 from *Ae. crassa*, and CL148 from *Th. bessarabicum* (Table 3 and Table S2). CL185 is a pericentromeric repeat. Bright signals were found on two *Ps. libanotica* and *Ps. tauri* chromosomes. In addition, the studied species had two additional chromosomes with less intense hybridization signals of CL185 (Figure 2).

CL192. CL192 has a length of 339 bp and a 0.029% genome proportion. It shared a 100% identity with P523 from *Ae. tauschii* and a 76–83% identity with Afa family repeats such as pAs1, pTa-535, and RcAfa (Table 3 and Table S2). CL192 is present in both species. The signals are located pericentromerically on two chromosomes (Figure 2).

CL207. CL207 has a length of 657 bp and a 0.028% genome proportion. It shared a 90.5% identity with CL18 from *Ae. crassa* and the FAT element (Table 3 and Table S2). Both studied species have pericentromeric localization sites of CL207, but the signal intensity varies among chromosomes. In *Ps. libanotica*, three chromosomes have bright signals, and three chromosomes have less intense localization sites. *Ps. tauri* is characterized by the presence of two chromosomes with strong pericentromeric signals of CL207 and four chromosomes with fainter signals (Figure 2).

## 3. Discussion

Studying the repeatome in wild grasses is important for understanding the processes of speciation. In total, the structure of the repeatome and the percentage of different lineages of mobile elements in *Ps. libanotica* were very similar to those revealed in [50]. According to the analysis of the whole-genome sequences, the number of *PIF/Harbinger* reads in *Ps. tauri* was 1.4 times larger than that in *Ps. libanotica* (Table 1), which agrees with the data obtained from the copy number of Pong (belonging to *PIF/Harbinger*) [58]. According to Markova et al. (2015), the abundance of *PIF/Harbinger* is equal in *Ps. spicata* and *Ps. tauri* [58]. However, according to our data, *Ps. spicata* has 6 and 10.5 times fewer *PIF/Harbinger* reads compared to *Ps. libanotica* and *Ps. tauri*, respectively, which can probably be explained by the different accessions of *Ps. spicata*. In our previous study, we found that *Ps. spicata Angela* showed an overwhelming majority among the studied transposons of the Ty1/*Copia* family [43], which is consistent with the findings of this study. In the genome of *Ps. libanotica*, it had almost twice as many satellite sequences, while the genome of *Ps. tauri* showed a higher proportion of the *Athila* element. Thus, although the overall structure of the repeatome between these two *Pseudoroegneria* species is similar, there are also some differences.

Satellite repeats can be used to create chromosomal markers that enable a comparative analysis between species, establishing the degree of their genetic similarity. Among the nine repeats localized to the *Ps. libanotica* and *Ps. tauri* chromosomes, four (CL69, CL101, CL119, CL244) showed predominantly terminal and/or distal localization (Figure 1), while six showed mainly pericentromeric localization (CL82, CL89, CL168, CL185, CL192, CL207) (Figure 2). The predominant localization in pericentromeric and/or terminal repeats is characteristic of non-dispersed repeats identified in the St genome, as described in the literature. Terminal localization on the chromosomes of the St genome is typical for the St-96 and St-98 repeats from *Ps. libanotica* [50], St_2_-80 and pPlTaq2.5 from *Ps. libanotica* [45,59], and S159 from *Ps. stipifolia* [47]. Pericentromeric localization has been shown for CentSt, S17, and S170 from *Ps. stipifolia* [47,49]. STlib_117 signals from *Ps. libanotica* were visible in the centromeric and terminal regions [50]. Interestingly, the repeats identified in the present study did not show any similarity to any of the previously published repeats found in the St genome.

CL69 signals were observed on all the chromosomes in the terminal regions of *Ps. tauri* and *Ps. libanotica* (Figure 1). Repeats similar to CL69 also showed predominantly telomeric localization in *Triticeae* species (Table 2 and Table S1), such as CL239 from *Ae. crassa* on the chromosomes of *Ae. crassa* and *Th. bessarabicum* [51], oligo-6VS-57 from *D. villosum* on the chromosomes of *D. villosum* [60], and oligo-7E-744 from *Th*. *elongatum* on the chromosomes of *D. villosum* and *D. breviaristatum,* as well as on the St chromosomes of *E. dahuricus* [61,62]. Thus, the conservation and ancient origin of the listed repeats and CL69 can be assumed to stem from a common ancestral repeat.

CL101 signals of varying intensity were observed on three pairs of *Ps. tauri* chromosomes, but they were not detected in *Ps. libanotica* (Figure 1). The similarity of CL101 to other repeats found in the species of *Aegilops*, *Triticum*, *Elytrigia*, and *Dasypyrum* may also indicate its ancient origin. At the same time, the percentage identity with the oligo-7E-744, pSp1B16, CL239, and Spelt1 repeats did not exceed 80%. The chromosomal distribution of the CL101 homologues across the *Triticeae* genomes includes both terminal and interstitial localization [51,61,62,63,64]. Therefore, CL101 and its related repeats have a different evolutionary fate and distribution among species and chromosomes.

The strongest distal CL119 signals were observed in *Ps. libanotica* and *Ps. tauri* on one pair of chromosomes, and minor signals were observed in various regions of the remaining chromosomes (Figure 1). The localization of the CL119-like repeats in *Triticeae* species is characterized by distal, subtelomeric, and terminal localization on chromosomes (Table 2 and Table S1) [51,63,64,65,66]. These repeats have also been found on B chromosomes of rye and *Aegilops* [67,68], except for pAcPR5, which is distributed across all P genome chromosomes of *A. cristatum* [69]. It may be noted that both CL119 and similar repeats predominantly produce the strongest signals on one or more pairs of chromosomes, including B chromosomes. This may suggest their role in the specificity of chromosome recognition during cell division.

The CL244 repeat used in this study was previously found in the genome of *Ae. crassa* [51]. *Ps. tauri* and *Ps. libanotica* exhibited a similar type of hybridization, occurring terminally on the long arm of one pair of chromosomes (Figure 1). In our previous study, CL244 hybridized terminally on several chromosome pairs of *Ae. crassa*, *T. aestivum*, and *Th. bessarabicum,* while in the latter species, the signals were the strongest. Given the conserved nature of localization and its distribution in many species of *Triticeae*, as well as the similarity of the CL244 terminal repeat to the Spelt52.1 repeats from *Ae. Speltoides* [70], pSc200 and pSc7235 from *S. cereale* [71,72], and BSCL1 and DP4J27982 from *Th. bessarabicum* [66,73], it can be assumed that CL244 refers to ancient repeats that arose before the divergence of the hypothetical ancient genome into separate genomes.

All six pericentromeric repeats showed homology to the FAT repeat (Table 3 and Table S2). Most often, the FAT element exhibits “fuzzy hybridization” with greater hybridization in the proximal and pericentromeric regions of the D genome chromosomes in *T. aestivum*, as well as on the chromosomes of the C, D, N, M, S, and U genomes in various *Aegilops* species [74]. The FAT repeat on *Ps. spicata* chromosomes shows a dispersed pattern in the proximal region, with the most intense signal observed in one pair of chromosomes [46]. Furthermore, all the pericentromeric repeats identified in the current study, with the exception of CL192, exhibited similarity to the CL18 repeat from *Ae. crassa*. CL18 exhibited an uneven distribution along the length of the chromosomes of *Ae. Crassa*, *Th. Bessarabicum*, *T. aestivum*, and *Ae. tauschii*, with more intense hybridization in the proximal chromosome regions [51]. The same five repeats showed homology to ACRI_CL80, which is localized pericentromerically to the *A. cristatum* chromosomes [75]. Four pericentromeric repeats, CL168, CL82, CL185, and CL89, showed homology to the pericentromeric repeat P631, which we previously found in the genome of *Ae. tauschii* and is characterized by either a discrete pericentromeric signal in *Th. bessarabicum*, *Th. intermedium*, and *Ps. spicata* or dispersed with strong pericentromeric signals in wheat and rye chromosomes [76,77]. This difference in hybridization patterns can be explained by the occurrence of these sequences in a common ancestor in the pericentromeric region of *Triticum*, *Aegilops*, *Thinopyrum, Secale,* and *Pseudoroegneria*. The number and distribution of elements have changed during subsequent evolution, resulting in variations in hybridization patterns. Although the listed repeats are homologous to each other, some of them are dispersedly spread from the pericentromeric region to the proximal regions, such as FAT and CL18. Others are localized in the pericentromeric region, like the six repeats we found and ACRI_CL80. Additionally, some repeats, such as P631, exhibit unique distribution patterns across different species.

It is worth noting that although CL89 (658 bp) is 100% identical to P631 (317 bp) (Table 3), it has a greater length (Table 4). Similarly, the pericentromeric repeat CL192 (339 bp) is 32% smaller in size than the P523 repeat (501 bp), which we previously identified in the genome of *Ae. tauschii* and is localized pericentromerically in the Js chromosome pair of *Th. intermedium* [76]. Thus, 100% identity in these cases indicates the proximity of these repeats, but not a perfect match.

The repeats CL89, CL82, and CL192 were found to be similar to Afa family repeats such as pAs1 and pTa535 from *T. aestivum* [78], RcAfa from *Roegneria ciliaris* [79], and CL3 from *Ae. crassa* [51] (Table 3). The Afa family is commonly used for chromosome identification in the *Triticeae* tribe and typically results in the detection of multiple subtelomeric, proximal, and interstitial hybridization sites on chromosomes [44,51,73,80]. CL82, CL89, and CL192 showed only pericentromeric signals in *Ps. tauri* and *Ps. libanotica* (Figure 1 and Figure 2). Despite the sequence’s proximity to the Afa family, the localization pattern of the repeats presented here is significantly different from that of the Afa family. This difference may indicate a divergence of CL192 from the ancestral form that is common to the Afa family.

Interestingly, the pericentromeric repeats found in the St genome showed homology to repeats that are predominantly terminal or have terminal localization (Figure 2, Table 3 and Table S2), for instance, P720 from *Ae. tauschii* (CL89, CL82, CL192) [76,77,81], S5 from *Ps. stipifolia* (CL82, CL192) [47], and Oligo-1AS from *Ae. speltoides* (CL89, CL207) [64]. The presence of pericentromeric repeats in terminal heterochromatic blocks, and vice versa, is a well-known phenomenon [82,83]. This phenomenon may be associated with their functional role in the recognition and segregation of chromosomes during cell division, as well as the stabilization of chromosome structure.

The comparison of the localization of the identified repeats on the chromosomes of *Ps. libanotica* and *Ps. tauri* provides the following classification.

(i) Repeats with a nearly identical hybridization pattern: CL244, CL185, CL82, and CL192.

(ii) Repeats with a similar pattern of hybridization with some differences: CL69, CL207, and CL168.

(iii) Repeats with different patterns of hybridization, exhibiting variations in the number of chromosomes or hybridization sites: CL119, CL101, and CL89.

Thus, based on this classification and a comparison of the repeatome structure, we can conclude that *Ps. libanotica* and *Ps. tauri* are distinct, closely related species, each with unique patterns of satellite repeat distribution and distribution along chromosomes. This conclusion is supported by the fact that both studied species cluster together in molecular genetic studies and share similar morphological characteristics [52,53,54,55,56,57,58]. The chromosomal markers we have created could be valuable for conducting population studies of these species, as well as for evaluating their biodiversity and speciation. Notably, the brightest signals are CL101 and CL168 in *Ps. tauri* and CL207 in *Ps. libanotica*, which were observed on an odd number of chromosomes, which is typical for cross-pollinated species with a heterozygous genome [40,51]. Among the three groups presented, the second and third groups may be the most suitable for such studies, as they exhibited differences among the studied *Pseudoroegneria* species. From this perspective, the satellite repeats revealed here can be utilized to determine the evolutionary status among different *Pseudoroegneria* accessions. For this purpose, the developed chromosome markers are to be precisely localized to specific linkage groups using bulked Oligo-FISH, which is based on a mixture of single-copy sequences [84]. The St genome chromosome markers developed in the present research can be useful in studies of polyploid species that contain the St genome, such as *Thinopyrum*, *Elymus*, *Kengyilia*, and *Roegneria*, as well as in wide hybrids.

## 4. Materials and Methods

### 4.1. Plant Materials

The following plant material was used in the study: *Ps. libanotica* PI 228389, *Ps. tauri* PI 380652, and *Ps. spicata* PI 578855. All accessions are diploids with the genomic formula StSt and were kindly provided by the USDA-ARS Germplasm Resources Information Network (GRIN).

### 4.2. Sequencing and Bioinformatics Analysis

Genomic DNA was isolated by the CTAB protocol [79]. The quality and quantity of the isolated DNA were tested using Qubit 4 (Thermo Fisher Scientific, Waltham, MA, USA) and electrophoresis in an 0.8% agarose gel.

Shotgun sequencing libraries were synthesized using the Swift 2S Turbo DNA Library Kit (Swift Bioscience, Ann Arbor, MI, USA), and their quality was checked using MiSeq (Illumina, Inc., San Diego, CA, USA). Already converted bible libraries were sequenced on the DNBSEQ-G400 device (MGI Tech, Shenzhen, China). The initial amount of DNA used was 25 ng, and fragments of about 350 bp in size were indexed at both ends using the Swift 2S Turbo Unique Dual Indexing Kit (Swift Bioscience, USA). Sequencing was performed on Illumina NextSeq (Illumina, Inc., San Diego, CA, USA) using the NextSeq 500/550 Mid Output Kit v2.5 (llumina, Inc., San Diego, CA, USA).

The subsequent study of nucleotide sequences, the search for repetitive DNA sequences, and the identification of their uniqueness were carried out in accordance with the methodology described in [45]. The sequences of primers for the identified satellite repeat monomers are shown in Table 4.

### 4.3. Fluorescence In Situ Hybridization (FISH)

The fixation of the material and the preparation of cytological preparations from the root meristems were performed in accordance with the methodology presented in the article [80]. The probes were localized on *Ps. libanotica* and *Ps. tauri* chromosomes using fluorescent in situ hybridization (FISH) according to the protocol published in [81]. Detection was carried out using sreptavidin-Cy3 (Vector Laboratories, Peterborough, UK) and Anti-dig-FITC (Roche, Basel, Germany). After the hybridization of the probes, chromosomes were stained with DAPI in the Vectashield medium (Vector Laboratories, Peterborough, UK). The signals were visualized using a DFC 9000 GTC fluorescence microscope (Leica Camera, Wetzlar, Germany) and further processed in Adobe Photoshop (Adobe, Inc., San Jose, CA, USA).

## 5. Conclusions

In the present study, a comparative analysis of the whole-genome sequences of *Ps. tauri*, *Ps. libanotica*, and *Ps. spicata* demonstrated the overall similarity in their repeatome structures, with some individual peculiarities observed in the abundance of the *SIRE* (Ty1/*Copia*) retrotransposons, *Mutator* and *Harbinger* transposons, and satellites. Nine St-genome satellite repeats were identified based on the whole-genome sequences. Specifically, three repeats were found in the genome of *Ps. tauri* (CL 89, CL 185, and CL 192), and six repeats were found in the genome of *Ps. spicata* (CL69, CL82, CL101, CL119, CL168, and CL207). The chromosomal localization of the nine satellite repeats on the chromosomes of *Ps. libanotica* and *Ps. tauri*, as well as the CL244 repeat that was previously discovered in *Ae. crassa*, was performed. The physical localization of the repeats allowed for the classification of the satellite repeats into two groups: (1) primarily terminal and/or distal, including CL69, CL101, CL119, and CL244; and (2) mainly pericentromeric, including CL82, CL89, CL168, CL185, CL192, and CL207. Each group of repeats showed homology to sequences already known in *Triticeae* species, which, in general, have a similar localization. The obtained results demonstrate that despite the general similarity between the studied species, they also exhibit specific differences in terms of the structure of the repeatome and the localization of satellite repeats on chromosomes.

## Figures and Tables

**Table 1:** Repeatome quantitative composition based on RepeatExplorer2.

Type of DNA Repeat	*Ps. libanotica*		*Ps. tauri*		*Ps. spicata*	
	Reads	Summarized%	Reads	Summarized%	Reads	Summarized%
Unclassified_repeat (conflicting evidences)	0	43.38	0	39.82	966	48.76
|--rDNA	0	0.41	0	0.22	0	0.25
| |--45S_rDNA	0	0.37	0	0.18	2385	0.18
| | |--18S_rDNA	3710	0.13	2684	0.11	0	0
| | |--25S_rDNA	7109	0.24	1532	0.07	1538	0.07
| ′--5S_rDNA	1267	0.04	1062	0.04	1551	0.07
|--satellite	157,704	5.35	55,132	2.36	122,341	5.42
′--mobile element	0	37.62	0	37.24	0	43.05
|--Class_I	0	35.31	0	35.01	0	40.03
| |--LTR	187,564	35.25	108,460	34.96	137,616	39.98
| | |--Ty1/*Copia*	110	7.92	0	8.34	0	9.30
| | | |--*Ale*	493	0.02	0	0	0	0
| | | |--*Angela*	143,057	4.85	124,821	5.33	104,690	4.64
| | | |--*Bianca*	166	0.01	0	0	0	0
| | | |--*Ikeros*	1825	0.06	794	0.03	1172	0.05
| | | |--*SIRE*	80,047	2.71	64,755	2.77	99,682	4.41
| | | |--*TAR*	7413	0.25	4935	0.21	4685	0.2
| | | |--*Tork*	617	0.02	0	0	0	0
| | ′--Ty3/*Gypsy*	0	20.98	0	21.99	0	24.58
| | |--non-chromovirus	0	14.06	0	15.89	0	17.39
| | | |--*Athila*	305,327	10.35	288,182	12.31	263,871	11.69
| | | |--*Ogre*	3420	0.12	2465	0.1	23,767	1.05
| | | ′--*Retand*	105,876	3.59	81,421	3.48	104,927	4.65
| | ′--chromovirus	0	6.92	0	6.1	0	7.19
| | |--*CRM*	30,327	1.03	17,216	0.73	17,452	0.77
| | |--*Tekay*	173,721	5.89	125,519	5.37	144,859	6.42
| ′--LINE	1825	0.06	1276	0.05	1267	0.05
′--Class_II	0	2.31	0	2.23	0	3.02
| |--*EnSpm/CACTA*	59,033	2	45,535	1.95	62,034	2.75
| |--*MuDR/Mutator*	5277	0.18	1668	0.07	5504	0.25
| |--*PIF/Harbinger*	3464	0.12	4840	0.21	306	0.02
′--*Helitron*	237	0.01	0	0	0	0
|--plastid	38,982	-	40,246	-	16,336	-
′--mitochondria	6774	-	0	-	4362	-
Unclassified repeat (No evidence)	335,904	-	251,051	-	206,323	-

**Table 2:** Results of the homology search for new St-genome terminal satellite repeats with known *Triticeae* repeats.

Repeat	Species of Origin	NCBI Accession	Identity to New Satellites, %		
			CL69	CL101	CL119
Sc26c38_V112	*S. cereale*	KC243240.1	xxx **	xxx	74.2
AesTR-183	*Ae. speltoides*	MK283667.1	xxx	xxx	75.4
pTa-465	*T. aestivum*	KC290905.1	xxx	xxx	77.8
CL131	*Ae. crassa*	ON872663.1	xxx	xxx	79.0
pAcPR5	*A. cristatum*	KX390696.1	xxx	xxx	82.5
BSCL156-3	*Th. bessarabicum*	n/a *	xxx	xxx	84.8
CL149	*Th. bessarabicum*	ON872689.1	xxx	xxx	85.0
BSCL156-1	*Th. bessarabicum*	n/a	xxx	xxx	86.5
18-158	*Th. ponticum*	n/a	xxx	xxx	86.9
BSCL156-2	*Th. bessarabicum*	n/a	xxx	xxx	89.7
Oligo-1AL	*T. aestivum*	n/a	xxx	xxx	90.0
CL232	*Ae. crassa*	ON872668.1	xxx	xxx	94.7
CL211	*Th. bessarabicum*	ON872686.1	71.9	xxx	xxx
CL239	*Ae. crassa*	ON872677.1	82.4	71.4	xxx
oligo-6VS-57	*D. villosum*	n/a	92.5	xxx	xxx
oligo-7E-744	*Th. elongatum*	n/a	98.2	79.5	xxx
Spelt1	*Ae. speltoides*	AY117402.1	xxx	68.3	xxx
pSp1B16.1	*Ae. speltoides*	FJ594248.1	xxx	69.7	xxx
pSp1B16.3	*Ae. speltoides*	FJ617549.1	xxx	76.3	xxx
pSp1B16.4	*Ae. speltoides*	FJ617550.1	xxx	69.0	xxx
Tri-MS-6	*T. aestivum*	EF469549.1	xxx	69.9	xxx

* data is not available; ** no homology was revealed.

**Table 3:** Results of the homology search for new St genome pericentromeric satellite repeats with known *Triticeae* repeats.

Repeat	Species of Origin	NCBI Accession	Identity to New Satellites, %					
			CL82	CL89	CL168	CL185	CL192	CL207
Oligo-1AS	*A. speltoides*	n/a *	xxx **	67.7	xxx	xxx	xxx	67.7
StLIB98	*Ps. libanotica*	OL685354.1	76.8	xxx	xxx	xxx	xxx	xxx
oligo-7E-430	*Th. elongatum*	n/a	78.2	xxx	xxx	xxx	xxx	xxx
oligo-5D151	*T. aestivum*	n/a	81.1	xxx	xxx	xxx	xxx	xxx
S5	*Ps. stipifolia*	n/a	81.3	xxx	xxx	xxx	72.7	xxx
pTa-451	*T. aestivum*	KC290912.1	87.5	xxx	xxx	xxx	xxx	xxx
CL149	*Th. bessarabicum*	ON872689.1	94.4	xxx	xxx	xxx	xxx	xxx
pAcPR3	*A. cristatum*	KX390694.1	xxx	xxx	xxx	xxx	82.1	xxx
FAT	*T. aestivum*	DX374230.1	81.2	74.6	91.7	82.9	73.0	90.5
pAs1-4, oligo-pAs1-1, pAs1	*A. speltoides*	n/a	xxx	85,7	xxx	xxx	83.3	xxx
RcAfa	*Roegneria ciliaris*	n/a	xxx	xxx	xxx	xxx	82.9	xxx
oligo-pTa535-1	*T. aestivum*	n/a	xxx	xxx	xxx	xxx	76.2	xxx
CL3	*Ae. crassa*	ON872662.1	84.4	85.7	xxx	xxx	74.4	xxx
CL18	*Ae. crassa*	n/a	84.2	75.0	91.7	91.7	xxx	90.5
ACRI_CL80	*A. cristatum*	MG323513.1	94.4	70.4	78.0	74.1	xxx	67.1
CL193	*Ae. crassa*	ON872676.1	73.5	76.8	80.4	82.6	xxx	xxx
CL148	*Th. bessarabicum*	ON872688.1	xxx	72.1	76.4	78.5	xxx	68.75
P631	*Ae. tauschii*	MK256651.1	85.2	100,0	82.9	95.5	xxx	xxx
P523	*Ae. tauschii*	MK256655.1	77.2	xxx	xxx	xxx	100.0	xxx
P720	*Ae. tasuchii*	MK256649.1	94.4	80.6	xxx	xxx	72.9	xxx

* data is not available; ** no homology was revealed.

**Table 4:** Primer sequences for the tandem repeats.

Repeat	Primers	Monomer Length, bp
CL69	F: 5′-ACTACCTTTTCAAGCCACCGT-3′ R: 5′-GGAGGTCATATATGGAGACCTATTT-3′	178
CL82	F: 5′-TGACACCATGCCAAGTTTCAT-3′ R: 5′-GTGCATGTTTAGGTCCCATGC-3′	503
CL89	F: 5′-CACTGGGCACAACCAAAGTT-3′ R: 5′-ACAAAAGGGCTCCATGCACA-3′	658
CL101	F: 5′-TTAAGGATGGTTTGGGCAGC-3′ R: 5′-ACCACACGTCACTCTGAAACA-3′	177
CL119	F: 5′-CCTTTGACTTTCGCCGGAC-3′ R: 5′- CGACACGGAGGGAATCTTGC-3′	668
CL168	F: 5′-TTTTTGTGAAGCAAGTGCCAT-3′ R: 5′-TAGAGCACACTTGCAGTTCA-3′	476
CL185	F: 5′-CACATGGGATGCCAACTGC-3′ R: 5′-TGGTCGAAACTAGAGCACACT-3′	659
CL192	F: 5′-TATACGCCATTGGAAGCCCC-3′ R: 5′-ACTCGTTAGCACGCCCAAAT-3′	339
CL207	F: 5′-TTGGATGGCCACTGACCAAG-3′ R: 5′-TGGCAATTTTCAGGACCAAACT-3′	657

## Data Availability

Data are contained within the article or Supplementary Materials.

## Supplementary Materials

The following supporting information can be downloaded at https://www.mdpi.com/article/10.3390/plants12244169/s1. References [46,47,50,51,60,61,62,63,64,65,66,67,68,69,70,74,75,76,77,78,79,85,86,87,88,89,90,91] are cited in the supplementary materials. Supplementary Tables: Table S1: Results of the homology search for new St genome terminal satellite repeats with known *Triticeae* repeats. Table S2: Results of the homology search for new St genome pericentromeric satellite repeats with known *Triticeae* repeats. Figure S1: Proportion of repetitive DNA sequences in *Ps. libanotica*. Figure S2: Proportion of repetitive DNA sequences in *Ps. tauri*. Figure S3: Proportion of repetitive DNA sequences in *Ps. spicata*.
